# Accuracy of estimates of cumulative load during a confined activity: bicycling

**DOI:** 10.1080/23335432.2019.1642141

**Published:** 2019-07-21

**Authors:** Anthony A. Gatti, Monica R. Maly

**Affiliations:** a School of Rehabilitation Sciences, McMaster University; 1280 Main St. W., Hamilton, Ontario, Canada; b Department of Kinesiology, University of Waterloo; 200 University Ave, Waterloo, Ontario, Canada

**Keywords:** Cumulative load, cycling, validity

## Abstract

Cumulative load reflects the total accumulated load across a loading exposure. Estimated cumulative load can identify individuals with or at risk for pathology. However, there is no research into the accuracy of the estimated cumulative load. This study determined: (1) which impulses, from a 500 revolution bicycling activity, accurately estimate cumulative pedal reaction force; and (2) how many impulses are required to accurately estimate cumulative pedal reaction force over 500 revolutions. Twenty-four healthy adults (mean 23.4 [SD 3.1] years; 11 men) participated. Participants performed three bicycling bouts of 10-min in duration and were randomized to one of two groups (group 1 = self-selected power and prescribed cadence of 80 revolutions per minute; group 2 = prescribed power of 100 W and self-selected cadence). The first 10 revolutions (2%) of the normal pedal reaction force (PRF_N_) and resultant pedal reaction force (PRF_R_), and the first five revolutions (1%) of the anterior-posterior reaction force (PRF_AP_) over-estimated cumulative load. The PRF_N_, PRF_AP_, and PRF_R_ required 80 revolutions (16%), 320 revolutions (64%) and 65 revolutions (13%), respectively, to accurately estimate cumulative load across 500 cycles. These findings highlight that the context and amount of data collected are important in producing accurate estimates of cumulative load.

## Introduction

Cumulative loading is an important mechanism contributing to musculoskeletal injury and pathology (Kumar 1990). Cumulative load reflects the total accumulated load across a researcher-defined loading exposure (e.g. minutes, days, months, years). The first reported use of cumulative load was in the spine (Kumar 1990). Cross-sectional cumulative compressive and shear loads on the thoracolumbar and lumbosacral discs, calculated using a two-dimensional mathematical model, distinguished between institutional aids with versus without back pain (Kumar 1990). Cumulative spinal loads were greater in 104 automotive workers who reported low back pain, compared to 130 randomly selected controls from the same workplace (Norman et al. 1998). It is important to note that poor relationships exist between peak and cumulative loads, suggesting these variables are distinct (Norman et al. 1998). Since these seminal papers, cumulative load has been used to characterize low back injury (Newell and Kumar 2005; Gregory et al. 2006) and knee osteoarthritis (Maly et al. 2013). Furthermore, low cumulative load reflecting a lack of mechanical loading has been shown to be detrimental to musculoskeletal health (Hinterwimmer et al. 2004; Souza et al. 2012), highlighting that there is likely a range of optimal loading exposures needed for musculoskeletal health.

While cumulative load shows promise in understanding injury mechanisms, methods used to calculate these values introduce error. For example, to calculate cumulative load, musculoskeletal models are typically utilized to calculate the representative impulse of a single repetition of the movement of interest (Equation (1) or (2)) experienced at a joint. The mean impulse of a sample of

(1) $$J\;=\;\int_{t_{0}}^{t_{1}}F\;dt$$

(2) $$J\;=\;\int_{t_{0}}^{t_{1}}M\;dt$$

$$J=impulse\;\left(N*s\;or\;Nm*s\right),$$

$$F=force\;\left(N\right),\;M=moment\;\left(Nm\right),$$

$$t_{o}=starting\;time,t_{1}=finishing\;time$$

movements is then multiplied by the measured number of loading repetitions to yield cumulative load (Equation 3) for a single activity (Petersen et al. 2015), a work day (Gregory et al. 2006; Maly et al. 2013), a work year (Newell and Kumar 2005), or even an individual’s entire working

(3) $$C_{L}^{\;}\;=\;J\;*R$$

$$C_{L}=cumulative\;load,$$

R=repetitions

career (Ezzat et al. 2013). There are many sources of potential error in this approach, including in data collection and analysis (e.g. gait analysis overground versus a treadmill (Riley et al. 2008); active or passive motion capture, or digitized video (Fonda et al. 2014); filter cut-offs (Van Den Bogert and De Koning 1996; Giakas and Baltzopoulos 1997), and the method of determining the start and end of a trial (O’Connor et al. 2007)) that alter the loading measurement, and therefore impulse calculations. Furthermore, each musculoskeletal model used to calculate the impulse of a task relies on assumptions that alter load measurements. For example, the calculated spine load was significantly different between four different spine models (*η^2^* = 0.97, *p* < 0.05) despite the use of the same inputs (Fischer et al. 2007).

Not only do data collection methods introduce error when calculating cumulative load, but there is also error inherent in the assumption that all repetitions of a task are the same. For example, if the loading magnitude is highly variable, using a single impulse or mean of a small number of impulses may produce large errors in cumulative load estimates. Numerous characteristics may affect movement variability and the resulting load characteristics. For example, during submaximal tasks an individual may be free to use a greater variety of movement patterns, resulting in greater intra-participant variability that reduces the accuracy of cumulative load estimates.

Basic science research cannot attempt to decipher whether musculoskeletal conditions are ameliorated or worsened by peak loads, loading rates, cumulative loads, or more likely some higher-order interaction unless each can be measured with sufficiently small error. *In vitro* research has the advantage of enabling more direct load measurements (Parkinson and Callaghan 2007). However, *in vivo* analysis of long-term adaptations (Van Ginckel et al. 2010; Multanen et al. 2014) or acute responses (Eckstein 2005; Gatti et al. 2017) of musculoskeletal tissues to different loading conditions requires estimation of these parameters. Therefore, accurate methods of estimating cumulative load must be identified before cumulative load can be applied to answer these types of research questions.

This is a proof-of-principle investigation that explores how to minimize the magnitude of error in cumulative load estimates. Bicycling was explored because it is a highly constrained, cyclic task. The outlined methodologies may be used to validate estimates of cumulative load for other activities. Furthermore, establishing adequate accuracy of estimates of cumulative load during cycling is necessary for both clinical research of bicycling as an intervention and basic research of the effect of bicycling-related joint loading on tissue structures. Identifying accurate methods of estimating raw cumulative load is a necessary first step before the complex interactions between cumulative load, fatigue, loading rate, peak load, and others can be pursued.

The purpose of this study was to determine: (1) which impulses, across a 10-min bout of bicycling, can be used to accurately estimate cumulative pedal reaction force; and (2) how many impulses are required to calculate a mean that yields an accurate estimate of cumulative pedal reaction force. A secondary objective was to determine whether relative and absolute activity conditions (cadence and power) influenced the accuracy of estimates of cumulative load. It was hypothesized that impulses sampled during the acceleration phase of a bicycling bout will yield poor accuracy when estimating cumulative load because accelerating the bicycle from zero will require greater forces and durations per cycle than during a steady-state. Also, it was hypothesized that 10% of impulses collected during the zero-acceleration phase of the activity are required to accurately estimatecumulative load. Ten percent was selected as an estimate of a reasonable amount of data for the researcher to collect in order to predict the cumulative load of an activity. For the secondary objective, it was hypothesized that variations in relative power would reduce the accuracy of the estimated cumulative load because participants would have greater movement variability when bicycling under conditions other than self-selected.

## Methods

A cross-sectional experimental design was used in healthy adults to address the primary and secondary research questions. All participants provided written informed consent. This study was approved by the Hamilton Integrated Research Ethics Board (HIREB).

### Participants

Twenty-four healthy adults (mean 23.4 SD [3.1] years; mean 23.13 SD [2.94 kg/m^2^]; 11 men and 13 women) completed this study. Participants reported high Lower Extremity Functional Scale scores (mean 79.3 [SD 1.8]) indicating no impairments in lower extremity function (Wang et al. 2009). Participants were excluded if it was deemed unsafe for them to partake in exercise, as determined using the Physical Activity Readiness Questionnaire (PAR-Q)(Canadian Society for Exercise Physiology 2004).

### Protocol

Participants completed one study visit and wore shorts, a t-shirt, and running shoes. Participants completed all bicycling bouts on a research-grade cycle ergometer (Lode Excalibur Sport, Groningen, NL). The bicycle was fitted to each participant using commercial guidelines based on inseam measurement (Eric Bowen 2011; Gatti et al. 2015). Pedal straps secured participant’s feet to the pedals. After a 5-min warm-up, participants completed three 10-min bouts of cycling during which two-dimensional pedal reaction forces were collected. Because errors in estimating cumulative pedal reaction forces may vary based on power and cadence, participants were randomized into one of two groups (Table 1). Group 1 bicycled with a standard power output: 100 watts. The first bicycling bout was at a self-selected pedaling cadence; the remaining two bouts were at 10% more and 10% less than the self-selected cadence, presented in randomized order. Group 2 bicycled at a standard pedaling cadence: 80 revolutions per minute (RPM). The first bout was at a self-selected power output (watts), and the remaining two bouts were at a power 10% more and 10% less than self-selected power, presented in randomized order.

**Table 1.** Description of powers and cadences performed by participants in Groups 1 and 2. This table shows the three conditions for relative power and cadence (self-selected moderate, +10% and -10% of self-selected).

Group		1^st^ Bout	2^nd^ Bout	3^rd^ Bout
1	Power (W)	100	100	100
	Cadence (RPM)	Self-selected	+10%	−10%
2	Power (W)	Self-selected	+10%	−10%
	Cadence (RPM)	80	80	80

### Instrumentation and signal processing

**Figure 1.**
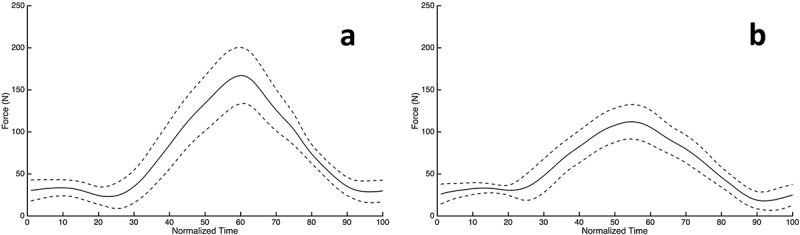
Representative force data from the first 500 revolutions of a single bicycling bout were extracted for one participant. Each of the 500 revolutions was time-normalized (0–100), and the mean (solid line) and 95% confidence interval (dashed line) of the time-normalized pedal reactions forces for the PRF_N_ (A) and PRF_AP_ (B) are plotted.

While pedaling, the normal (PRF_N_) and anterior-posterior (PRF_AP_) pedal reaction forces were measured in newtons (N) at 1kHz using a custom load measuring bicycle pedal (Novatech, East Sussex, UK) attached to the right bicycle crank arm; an equivalently shaped and weighted, but non-functional, apparatus was attached to the left crank arm. The load measuring pedal measured down and anterior as positive for the PRF_N_ and PRF_AP_. Data were collected and analyzed for the entire pedal revolution. The collected pedal reaction force data (PRF_N_, PRF_AP_) were filtered using a dual-pass, second-order, low-pass Butterworth filter at 10 Hz, which was determined using residual analysis (Winter 2005). An example of the filtered PRF_N_ and PRF_AP_ data for a single participant are presented in Figure 1. The resultant pedal reaction force (PRF_R_) was calculated as the resultant of the filtered PRF_N_ and PRF_AP_ force data. Data collected from a hall effects switch (Allegro Microsystems, Worcester, USA) were used to count the number of revolutions, and to separate force data into individual revolutions. The time integral of PRF_N_, PRF_AP_, and PRF_R_ (i.e. the respective impulses) were calculated for each of the first 500 revolutions for each participant.

### Outcome measurements

The gold standard measure of cumulative load was the sum of the measured cumulative PRF_N_ and PRF_AP_, as well as the PRF_R_ of the first 500 revolutions of each participant’s activity. Five-hundred revolutions were used because all participants completed at least 500 revolutions in all trials. The first 500 cycles were used for all participants. All cycles, including potential acceleration and deceleration cycles, were included in order to explicitly identify the number of revolutions that should be omitted for these purposes.

Estimates of cumulative load were produced for all force measurements (estimated PRF_N =_ ePRF_N_, estimated PRF_AP_ = ePRF_AP_, estimated PRF_R_ = ePRF_R_) by multiplying the mean of the revolutions of interest by 500 (total revolutions analyzed for each participant). For objective 1 (i.e. which impulses), the mean of 5 revolutions (1% of the data), extracted in 100 moving windows from the first to the 100^th^ percent, were used to calculate estimated cumulative load. For objective 2 (i.e. how many impulses), estimates of cumulative load were produced using a growing sample of the remaining data. Each sample used 1% more data than the previous sample. The growing sample excluded the impulses that were deemed to be inappropriate for estimation purposes during objective 1.

### Statistical analyses

Least squares regression and root mean squared error (RMSE) were used to analyze the PRF_N_, PRF_AP_, and PRF_R_ cumulative load data. In the regression analyses used to complete Objectives 1 and 2, regression through the origin (RTO)(Eisenhauer 2003) was used. RTO forces an intercept of 0 and was used to improve the interpretability of the fitted models. This approach avoids the challenge of interpreting variations in both intercept and slope simultaneously. RTO is also theoretically appropriate for assessing the relationship between the measured and estimated cumulative loads because they should have an intercept of 0. All reported RMSE values were normalized to the mean total impulse of all participants and represent percent error. All described methods were used for the PRF_N_, PRF_AP_, and PRF_R_.

#### Objective 1: which impulses accurately predict cumulative load?

Regression and RMSE analyses were completed between measured cumulative load and the estimate produced using each individual percent. To identify outliers, 95% confidence intervals were used. If any percent fell outside of the 95% confidence interval for the mean slope, R^2^, and RMSE of all 100% of the data, it was deemed to be an outlier and excluded.

#### Objective 2: how many impulses needed to accurately predict cumulative load?

The number of data points required to ensure RMSE was < 5% was identified. Prior studies have showed differences in cumulative load between healthy and clinical populations in the range of 7–15% (Norman et al. 1998). An RMSE of 5% is equivalent to a(minimum detectable difference at 95% confidence of ± 13.85% ($minimum\;detectable\;difference=\;RMSE\;\times \;z-score\;of\text{\textbackslash{}break}desired\;confidence\;\times \;\sqrt{2})$. Therefore, an RMSE of <5% will enable identification of differences of >14% at the individual level, with smaller differences detectable for group statistics. The slope and R^2^ were also determined and are presented for completeness. All the above analyses were performed using the Statsmodels module in Python 2.7.

#### Secondary objective: effect of activity intensity on cumulative load prediction

Relative cadence and power (1 = self-selected moderate, 2 = self-selected +10%, 3 = self-selected -10%), as well as absolute cadence (RPM) and power (W), were used to investigate this secondary objective. The effect of absolute and relative cadence on estimating cumulative load was tested using the samples of data determined in steps 1 and 2 to produce an accurate estimate of cumulative load for each of the PRF_N_, PRF_AP_, and PRF_R_. **Relative Cadence and Power**: Analysis of variance (ANOVA) was used to determine whether the error in estimated cumulative load (error = estimated – measured) was different between the three relative efforts (self-selected, +10%, -10%). In total, six ANOVAs were run. Two ANOVAs were conducted for each of the three force measurements (PRF_N_, PRF_AP_, PRF_R_); one ANOVA for each of cadence and power. Note that explicit corrections for conducting multiple tests were not conducted. **Absolute Cadence and Power**: Three regressions were run, one for each pedal reaction force, with the error in estimated cumulative load as the dependent variable and absolute cadence and power as well as participant as predictors. The regression variance-covariance matrix and standard errors were adjusted to allow multiple observations per participant using the vce(cluster) command in Stata 13.1. All statistical analyses associated with this secondary objective were performed using Stata 13.1 (StataCorp LP, TX, USA).

## Results

The mean power output for all trials (3 per participant) was 105.0 (SD 27.5) W, and the mean cadence was 78.6 (SD 8.0) RPM. The mean self-selected cadence (Group 1) was 77.3 (SD 9.0) RPM, and the mean self-selected power output (Group 2) was 110.0 (SD 36.7) W. Half of all participants (12/24) were randomized to Group 1 (constant power). There were no statistical differences (*p*> 0.350) in the measured cumulative load between these Groups for any of the three reaction forces (Difference [95% CI]: PRF_N_ = -1445.3Ns [-6115.8 to 3225.3], PRF_AP_ = -1412.5Ns [-4456.5 to 1631.5], PRF_R_ = -2130.4Ns [-6788.4 to 2527.7]).

Descriptive statistics of the measured cumulative load, by group, are presented in (Table 2). The coefficient of variation (*cv = σ ÷ μ* where: *cv*= coefficient of variation; σ = standard deviation; μ = mean) of measured impulses was calculated. On average, the *cv* of the PRF_N_ and PRF_R_ was 13.9% and 13.5%, respectively; the PRF_AP_ *cv* was 23.7%. There was a statistically significant difference in *cv* between PRF_AP_ and both PRF_N_ **(p < 0.001**; difference = -9.8%; 95% CI -12.7% to -6.9%) and PRF_R_ (**p < 0.001**; difference = -10.2%; 95% CI -13.1% to -7.4%).

**Table 2.** Mean and standard deviation of measured cumulative reaction forces (total impulse) for the PRF_N,_ PRF_AP_, and PRF_R_ groups, and both groups combined. There were no statistically significant differences in measured cumulative load between the groups, for any of the three reaction forces (p > 0.35).

Force Direction	Mean Cumulative Load by Group		
	Group 1 *Constant Power*	Group 2 *Constant Cadence*	Both Groups Combined
PRF_N_	41,951.56 (9008.9) Ns	40,506.3 (10,782.4) Ns	41,228.9 (9891.9) Ns
PRF_AP_	13,757.6 (4453.2) Ns	15,170.1 (8001.89) Ns	14,463.8 (6468.8) Ns
PRF_R_	45,966.9 (10,500.5) Ns	43,836.5 (9279.5) Ns	44,901.72 (9897.1) Ns

### Objective 1: which impulses accurately predict cumulative load?

**Figure 2.**
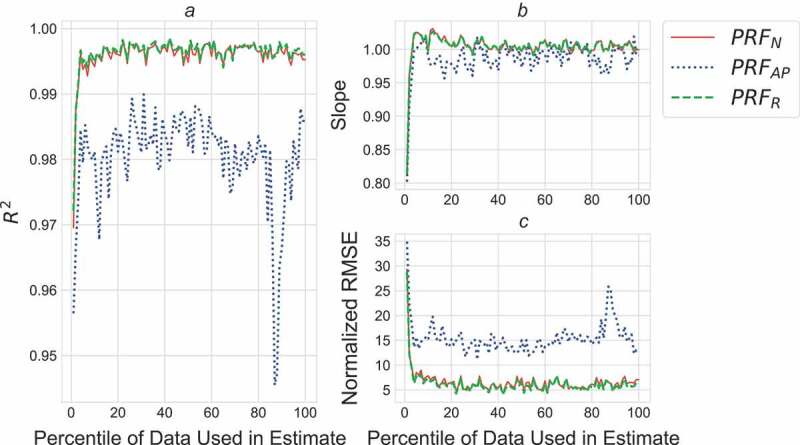
R^2^ (A), slope (B), and normalized RMSE (C) for the following estimates of cumulative load: ePRF_N_ (solid red), ePRF_AP_ (dotted blue), and ePRF_R_ (dashed green) produced using each individual percent of the collected data.

All but the first 10 revolutions (2%) of the data fell within the 95% confidence interval for R^2^, slope, and RMSE and were thus deemed suitable to estimate the PRF_N_ and the PRF_R_ (Figure 2). Only the first five revolutions (1%) of the data were excluded for the PRF_AP_. As can be seen in Figure 2(b), the slope of the regressions used to predict measured cumulative load for these first few percents are <1.0, indicating that estimates produced using these data were overestimating cumulative load.

### Objective 2: how many impulses needed to accurately predict cumulative load?

**Figure 3.**
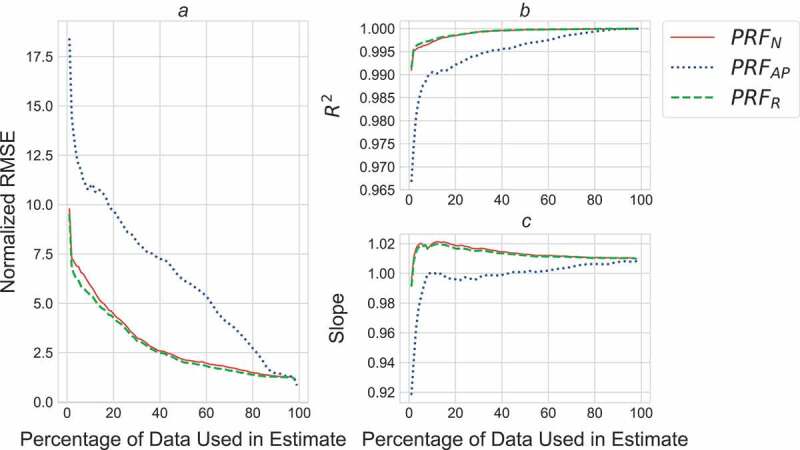
Normalized RMSE (A), R^2^ (B), and slope (C) for estimates of cumulative load produced using between 1% and 99% of the whole activity for PRF_AP_ (dashed blue), and between 1% and 98% of the whole activity for PRF_N_ (solid red) and PRF_R_ (dashed green).

Once data from the beginning of each bout (2% for PRF_N_ and PRF_R_; 1% for PRF_AP_) were excluded, using 80 revolutions (16%) of the PRF_N_, 65 revolutions (13%) of the PRF_R_, and 320 revolutions (64%) of the PRF_AP_ data resulted in an RMSE below 5% (Figure 3).

### Secondary objective: effect of activity intensity on cumulative load prediction

All comparisons of estimates of cumulative load for cadence and power were done using the 3^rd^ to 18^th^ percent (80 revolutions; 16%) of the data for the PRF_N_, the 2^nd^ to 66^th^ percent (320 revolutions; 64%) of the data for the PRF_AP_, and the 3^rd^ to 16^th^ percent (65 revolutions; 13%) of the data for the PRF_R_.

#### Relative power and cadence

Absolute error in estimates of cumulative load was not different between relative powers (PRF_AP_: R^2^ = .075, *p* = .277; PRF_N_: R^2^ = .032, *p* = .581; PRF_R_: R^2^ = .051, *p* = .427). There were no differences in absolute error between relative cadences (PRF_AP_: R^2^ = .047, *p* = .452; PRF_N_: R^2^ = .025, *p* = .658; PRF_R_: R^2^ = .031, *p*= .597).

#### Absolute power and cadence

For the PRF_AP_, neither power (*p*= .522) nor cadence (*p* = .142) were predictors of the error in cumulative load (R^2^ = .082, participant *p* = .217). For the PRF_N_, power (*p* = .052) and cadence (*p*= .062) approached but were not significant predictors of the error in cumulative load (R^2^ = .354, **participant *p* = .001**). For the model predicting the PRF_R_ error (R^2^ = .361, **participant *p* = .002**), power was approaching statistical significance (*p*= .064), but cadence was not (*p* = .343). To determine whether the inherent relationships between power and cadence with pedal force, and therefore cumulative load, were the cause of the borderline significant relationships observed, the error was normalized (difference/measured cumulative load) and regressions re-run. Normalizing the error increased the probability that there was no relationship (increased p – value) in all cases, except for cadence in the PRF_R_ model where cadence became a significant predictor of PRF_R_ error (PRF_N_: R^2^ = .275, power *p* = .134, cadence *p* = .217, **participant *p* = .002**; PRF_R_: R^2^ = .289, power *p* = .111, **cadence *p* = .033, participant *p* = .004**; PRF_AP_: R^2^ = .130, power *p*= .296, cadence *p* = .096, participant *p*= .080).

## Discussion and implications

This investigation determined that at least 65 revolutions of bicycling would be needed to make a valid estimate of the cumulative pedal reaction force of a 500 pedal-revolution (~10 min) bicycling bout in a sample of young, healthy adults. Furthermore, it was found that the first 1–2% of the collected bicycling data from 500 revolutions did not yield impulses that produce an accurate estimate of cumulative load in cycling. Greater accuracy of cumulative load estimates was achieved for measurements with low variability relative to the mean (i.e. PRF_N_) compared to those with large variability relative to the mean (i.e. PRF_AP_).

When estimating cumulative pedal reaction force from a bout of bicycling, the data collected at the start of this bicycling activity are not representative of the rest of the activity. In particular, the first 10 revolutions of the PRF_N_ and PRF_R_ and the first five revolutions of the PRF_AP_ from 500 revolutions yielded unacceptable estimates of cumulative load in cycling. These impulses at the beginning of the bicycling activity are on average greater than the impulses of the rest of the activity. As participants accelerated the pedal from rest, they were pedaling at a slower frequency than the rest of the activity; because power was held constant, they were, therefore, pedaling against greater resistance. The longer duration and larger resistance of these first revolutions led to necessarily greater impulses. These findings highlight that the context under which data iare collected can affect the accuracy of the estimated cumulative load. In this case, revolutions from the start of an activity are not representative of the rest of the activity. This finding is useful to consider when interpreting running biomechanical analyses. For example, it has been reported that overground and treadmill running kinetics and kinematics are different (Riley et al. 2008). In the investigation by Riley et al., overground running was down a 15-m runway and required acceleration and deceleration of the runner. Conversely, treadmill running allowed a 3–5-min warm-up followed by 5 x 30s collections, resulting in data collection during a phase of constant velocity. It is likely that achievement of a truer steady state during treadmill running contributed, at least in part, to systematic differences between overground and treadmill analyses.

After eliminating impulses collected during the acceleration phase of each bicycling bout, it was found that the PRF_N_ and PRF_R_ required a modest number of revolutions to make a valid estimate of cumulative load with samples of 16% (80 revolutions) and 13% (65 revolutions) of the whole 500 revolution activity. On the other hand, the PRF_AP_ required a much larger 64% (320 revolutions) of the activity. It is likely that the larger number of collected revolutions needed to estimate cumulative load of the PRF_AP_ is due to the larger relative variability in the PRF_AP_ impulses. The larger relative variability could in part be explained by the fact that the PRF_AP_ forces and impulses are smaller than those for the PRF_N_. Furthermore, deviations in PRF_AP_ and PRF_N_ may have counterbalanced one another, yielding a more consistent resultant force profile. That is, from revolution to revolution, an individual has a relatively constant PRF_R_ force profile, though they may alter their cycling pattern in such a way that contributions from the PRF_N_ and PRF_AP_ forces change slightly. This counterbalancing can be thought of similar to Winter’s (Winter 1984) description of how the sagittal plane moments about the ankle, knee, and hip may vary, but their sum, which is referred to as the support moment, stays relatively constant. These findings indicate that given a common quantity of sampled data, estimation of PRF_R_ is more likely to be accurate.

The secondary analysis determined that there was no systematic effect of relative cadence or power on the absolute error in estimates of cumulative load for PRF_N_, PRF_AP_, or PRF_R_. After normalizing the error in estimates of cumulative load, absolute cadence was a significant predictor of the PRF_R_. The small magnitude of explained variance (R^2^ = .289) indicates that this finding is likely unimportant. Further, it is important to consider that conducting six regression analyses elevated the likelihood of finding a significant result. A Sidak correction for multiple comparisons would decrease the necessary level of significance to 0.0085, which would eliminate the statistical significance of this finding.

The results from this investigation suggest future studies must include tens to hundreds of impulse measurements to accurately estimate cumulative load. At least 65 revolutions of the activity must be used to make a valid estimate of cumulative load in cycling. When including the acceleration phase, at least 75 revolutions (15%) would need to be sampled. In comparison, estimates of the cumulative knee adductor moment during gait (Maly et al. 2013) used five trials and estimates of low back loading (L4/L5) during sheep-shearing (Gregory et al. 2006) used six trials. These activities (gait and sheep-shearing) are inherently different to bicycling, and therefore the results of this study do not translate directly. However, results from the present investigation highlight the need to determine whether these samples of data (five or six trials) are capable of yielding accurate estimates of cumulative load. Estimates from activities other than bicycling could require more or less data to yield a valid estimate of cumulative load. For example, it could be assumed that most individuals take thousands of steps a day meaning that they are more trained in walking than bicycling. This training may lead them to have more consistent movement patterns, and therefore loads. Nonetheless, bicycling is a task constrained by the bicycle itself, likely minimizing variability in force profiles used to achieve the task.

Furthermore, the estimates of cumulative load exposures during occupational tasks and gait reported in other investigations reflect activities performed outside of the laboratory (Gregory et al. 2006; Maly et al. 2013). Uncontrolled environments likely result in even greater variability in movement patterns, likely increasing error in these estimates. For example, outside of the laboratory individuals may walk at different speeds, or carry an item (e.g. purse) while walking, causing changes to ground reaction forces and the associated variability (Hsiang and Chang 2002). For cumulative load to be appropriately utilized for identifying cause and effect in musculoskeletal conditions, setting safe working limits, and providing exercise or clinical recommendations, there must be accurate and reproducible methods of measurement. The described methodologies provide guidelines to estimate cumulative load during a confined bicycling task. To enable accurate estimates produced during highly variable leisure time bicycling, walking, running, lifting, and carrying it is likely that new methods are needed. Use of inertial measurement units (IMUs) is likely an inexpensive and fruitful next step that will improve estimates of cumulative load for a range of free-living conditions (Ryan 2006; Skotte et al. 2014; Shull et al. 2014).

Future investigations that use cumulative load are encouraged to perform analyses of the accuracy of cumulative load estimates as technological advancement allows. For example, gait data continuously sampled on an instrumented treadmill could provide some insight into cumulative load estimates for gait. In such an investigation, similar analyses to those reported here could be employed to determine which portions of the collected walking bout are suitable to estimate cumulative load, and how many collected steps should be used. By performing these studies and likely improving accuracy, it is possible that stronger associations will be found, resulting in better discrimination between pathologic populations using cumulative load estimates.

### Limitations

These estimates were in a healthy young sample under controlled laboratory conditions and therefore represent a minimum sample size. When applied to the real world, and in clinical populations, it would be reasonable to expect there to be greater error, and therefore a greater number of collected revolutions are likely needed to make an accurate estimate of cumulative load during bicycling. Furthermore, a limited range of powers (63–220 W) and cadences (64–99 RPM) were tested and participants were instructed to maintain the same cadence throughout their activity. While these powers and cadences reflect a relatively broad range, these are lower than those that are often used by competitive athletes.

### Conclusion

When estimating cumulative pedal reaction forces of a bicycling activity, it is necessary to exclude data from the acceleration phase of the activity. For the normal and resultant forces, relatively few samples of data are needed to accurately estimate cumulative load. When estimating cumulative load in the anterior–posterior direction, a larger sample of trials is needed to accurately predict actual cumulative load. These results highlight the fact that researchers should investigate the accuracy of estimates of cumulative load.
